# Risk factors for bovine tuberculosis in low incidence regions related to the movements of cattle

**DOI:** 10.1186/1746-6148-9-225

**Published:** 2013-11-09

**Authors:** M Carolyn Gates, Victoriya V Volkova, Mark EJ Woolhouse

**Affiliations:** 1 Epidemiology Group, Centre for Immunity, Infection and Evolution, School of Biological Sciences, University of Edinburgh, Ashworth Laboratories, Kings Buildings, West Mains Road, Edinburgh EH9 3JT, UK; 2 Department of Population Medicine and Diagnostic Sciences, College of Veterinary Medicine, Cornell University, Ithaca, NY 14853, USA

**Keywords:** Scotland, Cattle movements, Tuberculin test, Sensitivity, Specificity, Officially tuberculosis free

## Abstract

**Background:**

Bovine tuberculosis (bTB) remains difficult to eradicate from low incidence regions partly due to the imperfect sensitivity and specificity of routine intradermal tuberculin testing. Herds with unconfirmed reactors that are incorrectly classified as bTB-negative may be at risk of spreading disease, while those that are incorrectly classified as bTB-positive may be subject to costly disease eradication measures. This analysis used data from Scotland in the period leading to Officially Tuberculosis Free recognition (1) to investigate the risks associated with the movements of cattle from herds with different bTB risk classifications and (2) to identify herd demographic characteristics that may aid in the interpretation of tuberculin testing results.

**Results:**

From 2002 to 2009, for every herd with confirmed bTB positive cattle identified through routine herd testing, there was an average of 2.8 herds with at least one unconfirmed positive reactor and 18.9 herds with unconfirmed inconclusive reactors. Approximately 75% of confirmed bTB positive herds were detected through cattle with no known movements outside Scotland. At the animal level, cattle that were purchased from Scottish herds with unconfirmed positive reactors and a recent history importing cattle from endemic bTB regions were significantly more likely to react positively on routine intradermal tuberculin tests, while cattle purchased from Scottish herds with unconfirmed inconclusive reactors were significantly more likely to react inconclusively. Case-case comparisons revealed few demographic differences between herds with confirmed positive, unconfirmed positive, and unconfirmed inconclusive reactors, which highlights the difficulty in determining the true disease status of herds with unconfirmed tuberculin reactors. Overall, the risk of identifying reactors through routine surveillance decreased significantly over time, which may be partly attributable to changes in movement testing regulations and the volume of cattle imported from endemic regions.

**Conclusions:**

Although the most likely source of bTB infections in Scotland was cattle previously imported from endemic regions, we found indirect evidence of transmission within Scottish cattle farms and cannot rule out the possibility of low level transmission between farms. Further investigation is needed to determine whether targeting herds with unconfirmed reactors and a history of importing cattle from high risk regions would benefit control efforts.

## Background

The prevention and control of bovine tuberculosis (bTB) in domestic cattle herds remains an ongoing and costly challenge for many industrialized countries, including those that have achieved officially tuberculosis free (OTF) status [1,2]. Tuberculosis free status does not imply the complete absence of bTB, but is instead awarded to a territory where the incidence of newly confirmed positive cases has remained below a set threshold for a number of consecutive years, and appropriate surveillance systems are in place to detect any increase in disease frequency [3]. Since the majority of cattle infected with bTB show no detectable clinical signs, surveillance systems are generally based on *ante-mortem* testing of individual cattle using intradermal tuberculin tests at intervals determined by the perceived herd-level risk and *post-mortem* examination of all bovine carcasses at abattoirs for lesions consistent with bTB [1,4,5].

Both surveillance methods are considered good herd-level screening tools in regions where the prevalence of infected cattle in confirmed positive herds is generally high [6]. However, limitations in their sensitivity and specificity at both animal and herd levels have been highlighted as the main barrier to eradicating bTB from low incidence regions [7,8]. The single intradermal comparative cervical tuberculin test (SICCT) used in routine herd surveillance compares the sensitivity of individual cattle to bovine (*Mycobacterium bovis*) and avian (*M. avium*) mycobacterial antigens injected intradermally at separate sites on the neck. Depending on the relative degree of reaction to the antigens after 72 hours, animals may be classified as positive reactors, inconclusive reactors, or non-reactors [9]. The sensitivity for detecting infected animals ranges from 55% to 99%, but may be lower for cattle in the early stages of infection or for cattle experiencing physiological stress from pregnancy, concurrent illnesses, or poor management conditions [10-13].

Most animals with positive tuberculin skin reactions are slaughtered for local disease eradication as well as to confirm the presence of bTB through *post-mortem* observation of visible lesions or isolation of *M. bovis* from cultured tissue samples. Animals with inconclusive skin reactions may be re-tested up to three times at 2-month intervals to monitor changes in the degree of reactivity. In the majority of cases, no further evidence of bTB infection is found and regulatory officials are faced with the challenge of deciding whether these herds were truly infected with bTB and should be subject to the same local eradication measures as herds with confirmed bTB [14-16]. The specificity of SICCT is considered to be at least 99% [11,17], but may be affected by previous sensitization to *M. avium, M. paratuberculosis*, and other environmental mycobacteria as well as non-specific immunological responses to the injection itself [18].

With the difficulty in interpreting positive and negative SICCT results in low incidence regions, many tuberculosis free countries have found it more cost-effective to scale back routine herd testing programs in favour of targeted surveillance against cattle imported from endemic regions [1,4,5]. This approach has likely been effective in reducing the number of infected cattle entering low incidence regions due to the direct effects of testing on identifying infected cattle as well as the deterrent effects of extra testing on farmers’ decisions to purchase cattle from endemic regions [19]. However, these benefits may be partially offset by the opportunity for false negative cattle that pass the import testing to spread infection in the delay between importation and the possibility of detection through routine surveillance measures. This is especially true for countries that rely on slaughter inspection as the sole means of routine surveillance to identify infected cattle [20,21]. It has been estimated that standard food safety inspections at abattoirs miss up to 47% of cattle with visible tuberculosis lesions [22].

Using data from Scottish cattle herds in the period from 2002 to 2009 leading up to OTF recognition, this study first traced the origins of cattle identified as reactors in confirmed bTB positive herds detected through routine herd testing or found to have visible lesions at slaughter inspection to determine whether bTB transmission occurs within Scotland. The animal- and herd-level risks associated with previous cattle movements were then explored to investigate the potential role of herds with unconfirmed positive or inconclusive reactors in the epidemiology of bTB. Finally, a series of case-case and case–control comparisons were performed to identify herd demographic risk factors that may aid in the interpretation of unconfirmed or inconclusive routine intradermal tuberculin testing results.

## Methods

### Surveillance for bTB in Scotland

Scotland was awarded OTF status in September 2009 under the provisions of the European Union Council Directive 64/432/EEC, which require that the incidence of confirmed positive bTB herds has remained below 0.1% for at least 6 consecutive years, and appropriate surveillance systems are in place to detect newly infected herds. In the period from 2002 to 2009 leading up to OTF recognition, Scotland conducted surveillance through (1) routine herd testing (RHT) using the SICCT; (2) *post-mortem* examination of all bovine carcasses at slaughter for visible lesions consistent with bTB; and (3) post-movement testing of cattle imported from the Republic of Ireland or Northern Ireland, and from regions of England and Wales with high bTB incidence as determined by the frequency of RHT in the parish of origin.

During the study time period, RHT in Scottish cattle herds was conducted once every 4 years and included all female cattle that previously calved, bulls greater than 12 months of age unless exempted by a veterinarian, cattle greater than 6 weeks of age that may be used for breeding, and any cattle purchased since the last RHT. A small number of farms considered to be at increased risk of bTB, including those with regular intake of Irish cattle, were subject to annual testing. Cattle imported from Ireland were subject to post-movement testing throughout the study period. Post-movement testing for cattle imported from high incidence parishes of England and Wales was first introduced in September 2005. All post-movement tests must be carried out within 60 to 120 days of the animal arriving on the receiving Scottish farm, unless the animal is slaughtered or subject to RHT during that time period. Complementary pre-movement testing of the cattle moving from high incidence parishes of England and Wales was introduced in May 2006.

Cattle that react positively to SICCT on either RHT or post-movement testing are most often slaughtered to check for visible lesions and to collect tissue samples for bacteriological culture to confirm the presence of bTB infections. During the years analyzed, cattle that reacted inconclusively to SICCT in Scotland were re-tested up to two times to monitor for changes in the degree of reactivity. At the herd level, detection of a confirmed reactor through RHT (either through lesions or culture) or of a lesioned carcass at slaughter inspection triggers immediate animal-movement restrictions, testing of all cattle at 60 day intervals until no further reactors are disclosed, and testing of contiguous herds within a 3 km radius of the confirmed positive herd or trace-linked to the confirmed positive herd through animal movements. In some cases, the presence of unconfirmed positive or inconclusive reactors may also trigger follow-up testing if there is reason to suspect bTB in the herd, *e.g.* a known movement of cattle from a herd infected with bTB.

### Surveillance data

The results from all *ante-mortem* bTB tests and all suspected or confirmed cases identified through slaughter surveillance were collated in the VETNET database operated by the UK Animal Health and Veterinary Laboratories Agency (AHVLA). Negative results for a herd were typically reported *en masse* with summary information on the number of cattle tested, total number of animals in the herd, date and type of test, herd production type, and administrative information for the farm including its unique county-parish-holding (CPH) identifier and geographic coordinates. In cases where a positive reactor, inconclusive reactor, or lesioned animal was identified, the animal’s passport number was entered into the VETNET database along with any follow-up test results or actions taken. For herds with multiple reactors, information on whether visible lesions were observed at *post-mortem* examination or *M. bovis* was cultured from individual reactors was aggregated at the herd level.

An extract of the VETNET database containing all bTB surveillance records for Scottish cattle herds from 01 Jan 2002 to 31 Dec 2009 was obtained from the Animal Health branch of the UK Department for Environment, Food and Rural Affairs (DEFRA). This analysis focused on the subset of 12,248 beef, beef fattening, beef suckler, and dairy herds with at least one recorded RHT observation during the study period, and also herds with lesioned animals detected through slaughter inspection (slaughter case) during this time. The RHT results from herds where testing was staggered across multiple dates for logistical reasons were aggregated into a single RHT observation for the calendar year preserving the median testing date for negative herds and the date where the first positive and/or inconclusive reactor was identified in herds with non-negative RHT results.

Each RHT observation was initially classified into one of four groups based on the aggregate test results reported in VETNET: (1) confirmed positive RHTs - where at least one SICCT reactor or inconclusive reactor was identified and subsequently confirmed to have bTB through visible lesions at *post-mortem* or culturing *M. bovis*, (2) unconfirmed positive RHTs - where at least one positive SICCT reactor was identified, but never confirmed to have bTB by observation of lesions or positive culture), (3) unconfirmed inconclusive RHTs - where only inconclusive SICCT reactors were identified and never confirmed to have bTB by observation of lesions or positive culture, and (4) negative RHTs - where no cattle reacted positively or inconclusively to SICCT. Over 98% of the unconfirmed positive RHT observations in the VETNET database triggered precautionary disease eradication measures, whereas only 5.7% of the unconfirmed inconclusive RHT observations triggered precautionary disease eradication measures. The passport numbers for all individual cattle identified as positive or inconclusive reactors on RHT and all cattle with lesions identified through routine slaughter inspection were extracted.

For reference, definitions of the bTB herd status classifications and terminology used to describe movement history of imported cattle are provided in Table 1.

**Table 1:** Definitions of the bTB herd risk classifications and terminology used to describe the movement history of imported cattle

**Term**	**Definition**
Herd risk classifications	
• Slaughter case	• A herd where at least one animal was found to have visible lesions consistent with bTB on routine slaughter inspection.
• Confirmed positive RHT	• An RHT observation where at least one SICCT reactor or inconclusive reactor was identified and subsequently confirmed to have bTB through visible lesions at *post-mortem* or culturing *M. bovis.*
• Unconfirmed positive RHT	• An RHT observation where at least one positive SICCT reactor was identified, but never confirmed to have bTB through visible lesions at *post-mortem* or culturing *M. bovis.*
• Unconfirmed inconclusive RHT	• An RHT observation where only inconclusive SICCT reactors were identified, but never confirmed to have bTB through visible lesions at *post-mortem* or culturing *M. bovis.*
• Non-negative RHT	• An RHT observation where at least one positive or inconclusive SICCT reactor was identified. This includes confirmed positive, unconfirmed positive, and unconfirmed inconclusive RHT observations.
• Negative RHT	• An RHT observation where no cattle reacted positively or inconclusively to SICCT.
Movement history categories	
• Overseas	• Cattle imported from the Republic of Ireland, Northern Ireland, or other overseas locations.
• High incidence parish	• Cattle imported from parishes of England and Wales with a testing interval of 12 or 24 months.
• Low incidence parish	• Cattle imported from parishes of England and Wales with a testing interval of 36 or 48 months.
• High risk import	• Cattle imported from overseas or from high incidence parishes of England and Wales.

### Cattle movement data

Records of the births, deaths, and movements of individual cattle in Great Britain have been stored in the electronic Cattle Tracing System (CTS) database operated by the British Cattle Movement Service (BCMS) since 1998 [23]. In this analysis, we used these records to trace the movement history of individual cattle present in the study herds prior to and on the date of RHT or slaughter inspection. The passport number, age, sex, and breed classification of each animal were also extracted from the CTS database. Locations within England or Wales were classified into bTB-risk groups based on the frequency of RHT for cattle farms within the parish. A high incidence region was taken to be one where the parish testing interval (PTI) was 12 or 24 months, and a low incidence region of was taken to be one where the PTI was 36 or 48 months. The list of PTIs published by DEFRA in 2007 was used to determine the location risk. Imports from the Republic of Ireland, Northern Ireland, and other overseas locations were grouped together under overseas imports. From this point forward, the term ‘high risk imports’ is used to refer to cattle imported to Scotland from overseas or from high incidence parishes of England and Wales.

Locations within Scotland were classified into one of eight bTB-risk groups based on their RHT history (confirmed positive RHT herd, unconfirmed positive RHT herd, unconfirmed inconclusive RHT herd, and negative RHT herd) as well as the presence of cattle imported from overseas and/or high incidence parishes (high risk imports or no high risk imports) at the time when studied cattle were located in the herd. The reason for further subdividing the locations by import movement history was that imported cattle are believed to be the primary source of bTB infections in low incidence regions [19]. A ninth category was also created for farm locations in Scotland with no RHT results reported in the VETNET database. These were most likely separately managed land parcels belonging to registered cattle businesses. Due to the infrequency of testing, all herds with confirmed and unconfirmed reactors were assumed to be at risk for spreading bTB in the 4 year period immediately before and the 4 year period immediately after testing.

The CTS database was also used to generate a list of farm locations (agricultural holdings and landless keepers) that housed cattle for at least one day between January 2002 and December 2009. Easting coordinates (increasing from west to east) and northing coordinates (increasing from south to north) within Great Britain for each location were obtained from either the VETNET database or the CTS’s Postal Address File. This information was used to calculate the total number of farms within a 5 km radius of each study herd and, through linking with the remainder of VETNET data, to determine whether at least one of those farms had (1) confirmed bTB positive cattle identified through RHT or (2) unconfirmed reactors or inconclusive reactors identified through RHT.

The total number of cattle present in Scotland, and the number of cattle from Scottish farms slaughtered at any abattoir in Great Britain from 2002 to 2009 were also recorded to provide descriptive statistics on surveillance coverage. It was assumed that all cattle slaughtered at an abattoir were subject to *post-mortem* examination for visible bTB lesions.

### Agricultural census data

Data from the annual June Agricultural Census of Scottish agricultural holdings provided by the Scottish Government were used to determine the average numbers of sheep and poultry present on the study farms in any given year from 2002 to 2009. These livestock species are potential reservoirs of cross-reacting mycobacterial antigens that may lead to false positive tuberculin test results in exposed cattle [18]. Sheep can also be infected with *M. bovis* and therefore represent a possible reservoir of bTB [24,25].

### Data quality issues

The VETNET, CTS, and June Agricultural Census databases each have different standards for recording information about individual cattle and individual herds. A number of observations were excluded from the analysis because the cattle passport number or the farm CPH code recorded in the VETNET database could not be linked to records in the CTS or Census databases. Descriptive statistics on the data linkage efficiency for individual reactors and individual herds by case type are presented in Table 2.

**Table 2:** Descriptive statistics on animal and herd data losses due to database linkage errors

	**Animals**		**Herds**			
		** CTS**		** CTS**	** Census**	** Both**
**Herd Status**	**N total**	**N matched**	**N total**	**N matched**	**N matched**	**N matched**
Slaughter case	61	54	49	46	44	44
Confirmed positive RHT	118	112	61	61	60	60
Unconfirmed positive RHT	207	198	124	124	119	119
Unconfirmed inconclusive RHT	1402	1320	843	841	790	790
Negative RHT	-	-	11171	11153	10061	9969
Total	1788	1684	12248	12225	11074	10982

The VETNET database was the starting data to which the CTS database and Census data were linked.

### Statistical analyses

Basic descriptive statistics on bTB surveillance in Scotland were first derived, and then a series of four statistical analyses was performed to investigate risk factors for disclosing tuberculin reactors on RHT. For all logistic regression models, the odds ratio (OR) and 95% confidence intervals (CIs) of the independent variables associated with the outcome were reported. Associations with a p-value < 0.05 were considered statistically significant. The cattle movement data were extracted from the CTS database using the Python programming language and all statistical analyses were performed in R software [26].

#### Animal-level risk associated with past movement history

The first analysis explored the animal-level risks of being identified as a positive or inconclusive reactor on RHT associated with past movement history. Due to the left censoring bias in the available surveillance and movement data, we chose to focus this analysis on records from 2006 to 2009. The study sample was further restricted to cattle that were located on at least one other farm prior to being tested through RHT. This was partly because we were interested in evaluating the risk of infection spreading from other herds within and outside of Scotland and partly because of the difficulties in determining which animals were subject to SICCT during the RHT from the aggregated results reported in the VETNET database. Based on the RHT selection criteria, all animals that were moved onto the farm since the last herd test should have been subject to testing. Therefore, we assumed that if no positive results were reported for the herd, then the purchased animals must have tested negative. The final sample contained 64 positive reactors, 294 inconclusive reactors, and 33,192 non-case animals.

A series of twelve binary categorical variables were created to classify the animal’s previous presence or absence in each of the following herd types: (1) overseas herd, (2) herd in a high incidence parish of England and Wales, (3) herd in a low incidence parish of England and Wales, (4) Scottish herd with a confirmed positive RHT and high risk imports, (5) Scottish herd with a confirmed positive RHT and no high risk imports, (6) Scottish herd with an unconfirmed positive RHT and high risk imports, (7) Scottish herd with an unconfirmed positive RHT and no high risk imports, (8) Scottish herd with an unconfirmed inconclusive RHT and high risk imports, (9) Scottish herd with an unconfirmed inconclusive RHT and no high risk imports, (10) Scottish herd with a negative RHT and high risk imports, (11) Scottish herd with a negative RHT and no high risk imports, and (12) Scottish herd with no records in VETNET.

A univariate analysis was initially performed to identify variables with a p-value < 0.20 for inclusion in the final multivariate model. The variables were screened individually using a multinomial logistic regression model with the outcome variable as the animal’s reactor status (negative, inconclusive, or positive). Further, we included age as a fixed predictor in all the ‘univariate’ and multivariate models as this is a potential confounder for being identified as a reactor on RHT. The variable selection for the final multivariate model was done as a backwards stepwise elimination process in which variables with the highest p-values were sequentially removed in turn until all the remaining variables in the model had a p-value < 0.05. Forwards stepwise selection was then performed adding in each of the eliminated variables in turn and checking for p-values of < 0.05 to ensure that none of the variables were wrongfully excluded based on the order of elimination during the backwards selection.

#### Herd-level risk associated with past movement history

The second analysis explored herd-level risk factors for having at least one reactor on RHT based on the history of cattle moved onto the farm in the 4 year period prior to the testing date. The analysis was again restricted to data from 2006 to 2009 because of the left censoring bias in the available surveillance and cattle movement data. For herds with multiple RHTs during this time period, a single observation was selected at random so that each herd appeared only once in the analysis. The resulting sample contained 18 confirmed positive RHT cases, 51 unconfirmed positive RHT cases, 327 unconfirmed inconclusive RHT cases, and 9,868 negative controls.

The same twelve binary movement history variables as described above for the animal-level risk factor analysis were used to characterize the origins of cattle purchased by the study herds and a similar approach was used for selection of the final multivariate models. However, in this analysis, we used a multinomial logistic regression model with the four RHT case types (negative, unconfirmed inconclusive RHT, unconfirmed positive RHT, and confirmed positive RHT) as the levels of the outcome variable. We also included the log transformed total number of individual cattle movements as a fixed predictor variable in all the ‘univariate’ and multivariate models as a potential confounder.

#### Comparisons of risk factors between confirmed positive, unconfirmed positive, unconfirmed inconclusive, and negative RHT herds

The third analysis used a series of case-case and case–control comparisons to explore difference in the demographic characteristics of herds that may aid in the interpretation of herd-level SICCT results. The independent variables included all data from 2002 through 2009 and were the (1) total number of unique animals present on the location, (2) percentage of unique animals that were imported from overseas or from high incidence parishes of England and Wales, (3) total number of intradermal tuberculin tests performed, (4) total number of cattle moved onto the farm, (5) total number of cattle farms within a 5 km radius, (6) presence of a herd with a confirmed positive RHT within a 5 km radius, (7) presence of a herd with an unconfirmed positive RHT observation within a 5 km radius, (8) presence of a herd with an unconfirmed inconclusive RHT observation within a 5 km radius, (9) easting coordinate (increasing west to east), (10) northing coordinate (increasing south to north), (11) herd type (categorized as beef, beef fattening, beef suckler, dairy, or mixed), (12) average number of sheep present on the farm per year, and (13) average number of poultry present on the farm per year. Variables 1, 3, 4, 5, 12, and 13 were log_10_ transformed prior to analysis. Variables 9 and 10 were expressed in units of 100 km. After excluding farms with missing data, there were 59 herds with confirmed positive RHTs, 120 herds with unconfirmed positive RHTs, 781 herds with unconfirmed inconclusive RHTs, and 9,972 herds with negative RHTs only.

Three case-case comparisons were initially performed using standard logistic regression: one comparing confirmed positive RHT herds to unconfirmed positive RHT herds, one comparing confirmed positive RHT herds to unconfirmed inconclusive RHT herds, and one comparing unconfirmed positive RHT herds to unconfirmed inconclusive RHT herds. Model selection was implemented as for the previous animal and herd-level analyses. The discrimination performance of the final models was assessed using the area under the receiver operating characteristic curve (AUC). A single case–control comparison was then performed using multinomial logistic regression with the four RHT case types (negative, unconfirmed inconclusive RHT, unconfirmed positive RHT, and confirmed positive RHT) as the levels of the outcome variable.

#### Change in herd-level risk of disclosing at least one tuberculin reactor over time

The final analysis explored changes in the risk of farms with recent high risk imports and farms with no recent high risk imports disclosing at least one positive or inconclusive reactor on RHT over time from 2002 to 2009. The objective was to determine whether the introduction of pre- and post-movement testing and subsequent changes in import movement patterns was associated with a decreased risk of having reactors. For the purpose of this analysis, confirmed positive RHT, unconfirmed positive RHT, and unconfirmed inconclusive RHT observations were combined into a single case group due to the low number of cases per year. Recent high risk imports were defined as having purchased at least one animal from overseas or from high incidence parishes of England and Wales during the 4 year period prior to the routine testing or slaughter inspection date. Two mixed-effects logistic regression analyses were performed with the year of bTB testing as the categorical fixed effect variable. The reference year was 2002. To correct for herds with multiple test observations over the 8 year period, the farm CPH code was included as a random effect in the model. In total, there were 595 cases with recent high risk imports used in the first model, 559 cases with no recent high risk imports used in the second model, and 22,986 negative RHT observations used as controls for both models.

## Results

### Descriptive statistics

**Figure 1:**
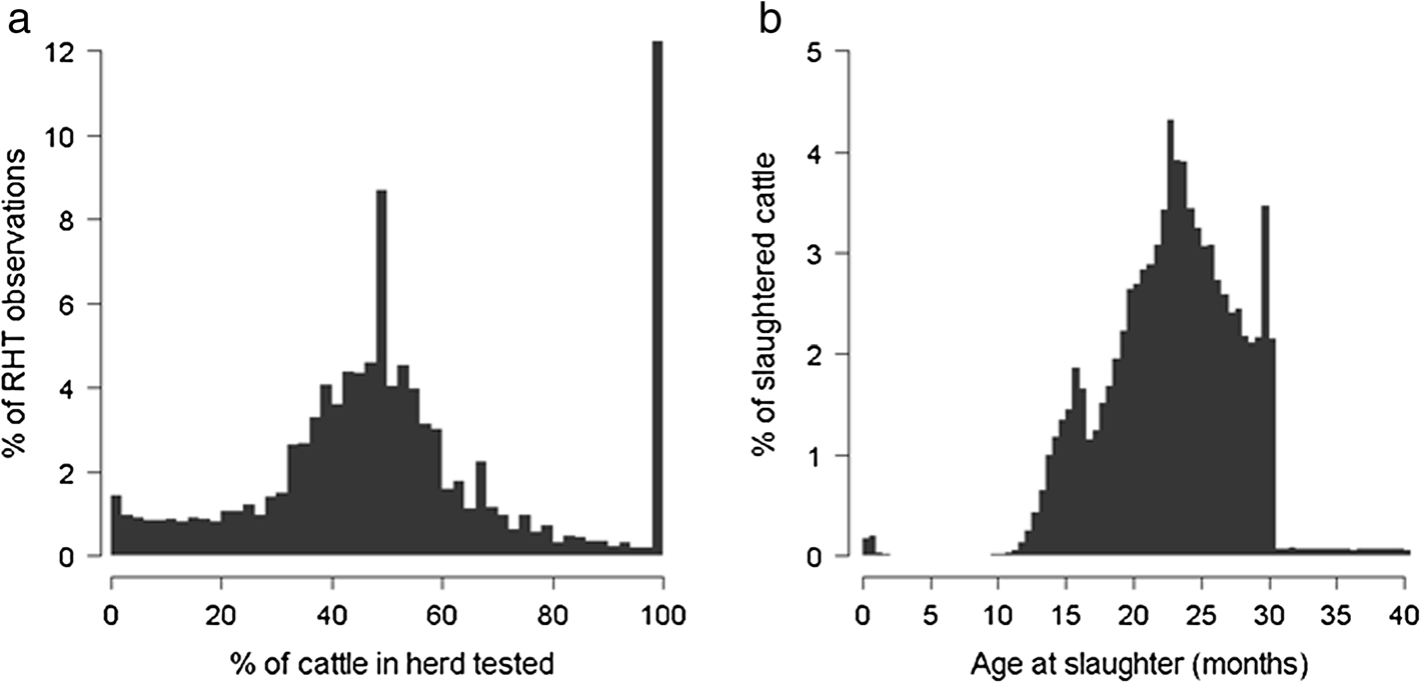
Distribution of the (a) percentage of cattle in Scottish herds tested during a single RHT observation and (b) the age of cattle at slaughter.

An estimated 7.25 million cattle were located on Scottish farms between January 2002 and December 2009 with an average of 1.92 million cattle present on any given day. During this time period, 4.36 million cattle were subject to *post-mortem* examination at abattoir and 1.68 million individual SICCTs were performed at RHTs with approximately 21% of all active cattle farms subject to RHT in a given year. As shown in Figure 1a, there was wide variation in the percentage of cattle in the herd that were tested during a single RHT. Approximately 85% of cattle were less than 30 months of age when slaughtered at abattoir (Figure 1b), reflecting the large proportion of Scottish cattle that were raised for beef production.

**Figure 2:**
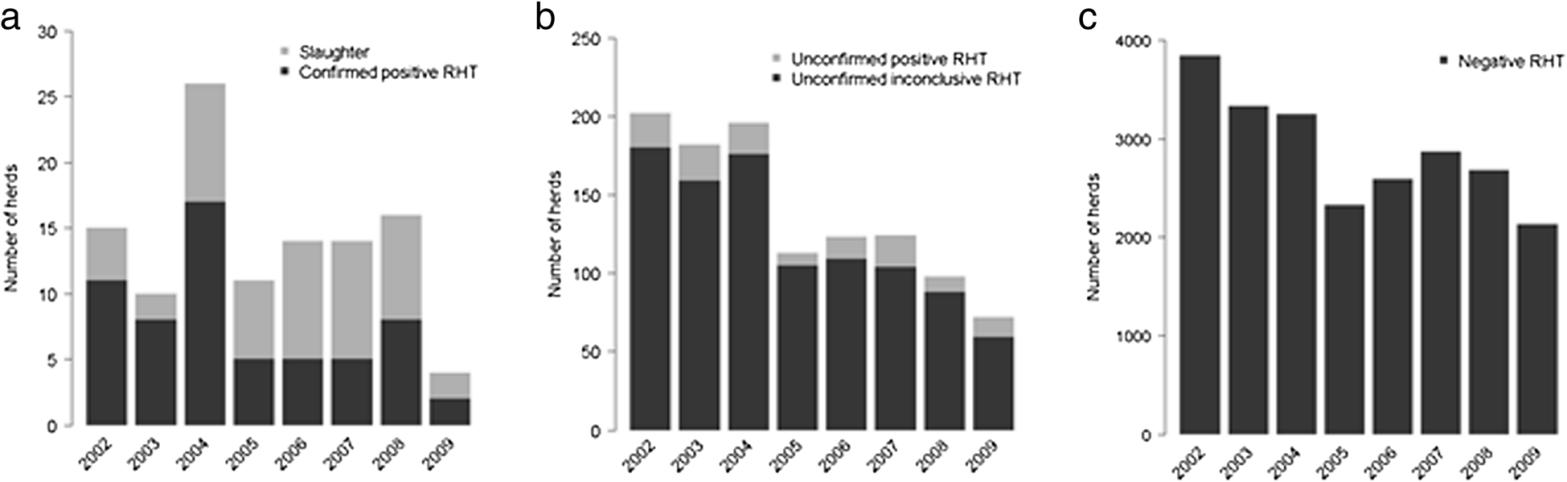
Frequency of bTB (a) slaughter cases and confirmed positive RHTs, (b) unconfirmed positive RHTs and inconclusive RHTs, and (c) negative RHTs in Scotland from 2002 to 2009.

Only 61 cattle were identified as having lesions consistent with bTB at routine slaughter inspection during the study time period. Of the cattle that were subject to SICCT through RHT, 267 were identified as positive reactors and 1,460 were identified as inconclusive reactors. Descriptive statistics on the frequency of slaughter cases, confirmed positive RHTs, unconfirmed positive RHTs, unconfirmed inconclusive RHTs, and negative RHTs are presented in Figure 2. For every confirmed positive RHT in any given year, there was an average of 2.8 unconfirmed positive RHTs (range: 1.2 to 4.0), 18.9 unconfirmed inconclusive RHTs (range: 10.4 to 21.8), and 489 negative RHTs (range: 190 to 1,064).

The average age of cattle identified as positive reactors on RHT was 6.8 years (median: 6.3, range: 1.0 to 17.1) and 93.7% were female cattle. Similarly, the average age of cattle identified as inconclusive reactors on RHT was 6.8 years (median: 5.9, range: 0.8 to 19.7) and 96.1% were female cattle. The average age of lesioned cattle identified through slaughter inspection was 7.9 years (median: 7.8, range: 1.5 to 17.5). A slightly lower proportion of lesioned cattle (85.2%) were female.

As shown in Table 3, the majority of cattle identified with lesions at slaughter or identified as positive or inconclusive reactors in confirmed positive RHT herds were either born in Scotland or located exclusively in Scotland for at least 4 years prior to the initial detection date. It is worth noting that approximately 70% of the animals with no known movements outside Scotland were at some point located on farms at the same time as cattle imported from overseas or from high incidence parishes of England and Wales.

**Table 3:** Import movement history of individual cattle identified as bTB lesioned animals at slaughter or as positive or inconclusive tuberculin reactors during RHT in Scotland from 2002 to 2009

	** Herd case type**							
	** Slaughter**		** Confirmed positive RHT**		** Unconfirmed positive RHT**		** Unconfirmed inconclusive RHT**	
	**N**	**%**	**N**	**%**	**N**	**%**	**N**	**%**
Scotland only	36	59.0	94	79.7	185	93.4	1159	87.8
Low incidence parish of England and Wales	1	1.6	0	0	0	0	2	0.2
High incidence parish of England and Wales	13	21.3	10	8.5	6	3.0	85	6.4
Overseas import	5	8.2	8	6.8	7	3.5	74	5.6
Missing data	7	11.5	6	5.1	9	4.5	82	6.2
Total	61	100	118	100	198	100	1320	100

Amongst the 103 confirmed bTB positive herds with complete movement history available for all positive animals identified on the initial RHT or slaughter inspection date, 47 of the 58 (81%) confirmed positive RHT herds and 31 out of 45 (72%) slaughter cases herds were identified through cattle with no known movements outside Scotland, providing indirect evidence that bTB transmission occurred within Scotland during the study time period.

In tracing the movement history of all cattle present in the case herds on the initial RHT or slaughter inspection date, 75 of the 107 confirmed positive RHT or slaughter case herds had at least one animal imported from overseas or from high incidence parishes of England and Wales (Table 4). In contrast, only 54.6% of unconfirmed positive RHT herds, 53.3% of unconfirmed inconclusive RHT herds, and 35.2% of negative herds had imported cattle present on the test or slaughter inspection date. However, the percentages were considerably higher when the movement history of the farms was traced further back in the CTS database.

**Table 4:** Presence of imported cattle in Scottish cattle herds subject to routine surveillance by bTB case type from 2002 to 2009

	**Case Type**									
	**Slaughter**		**Confirmed positive RHT**		**Unconfirmed positive RHT**		**Unconfirmed inconclusive RHT**		**Negative RHT**	
Presence of high risk imports:	N	%	N	%	N	%	N	%	N	%
On test date	33	71.7	44	72.1	71	54.6	521	53.3	8110	35.2
Four years prior	36	78.2	50	81.9	94	72.3	661	67.6	11492	49.9
All years prior	40	87.0	50	81.9	99	76.2	709	72.5	12664	55.0
Total matched herds	46	100	61	100	130	100	980	100	23010	100

The number and percentage of herds with imported cattle is shown. The movement history is sequentially expanded from cattle present on the test date to all cattle present up to 4 years prior to the test date and all cattle present in all years prior since inception of the CTS database in 1996.

### Animal-level risk associated with past movement history

In the subset of confirmed positive RHT, unconfirmed positive RHT, and unconfirmed inconclusive RHT herds from 2006 to 2009, there were a total of 118,066 animals (128 positive reactors, 535 inconclusive reactors, and 117,403 non-cases) present on the date when the suspect animals were initially identified. Of these, 33,550 animals (64 positive reactors, 294, inconclusive reactors, and 33,192 non-cases animals) were located on at least one other cattle farm prior to the test date with 34.3% located on 2 or more farms. Results from the univariate and multivariate analyses of movement based risk factors (Table 5) showed that cattle purchased from Scottish herds with unconfirmed positive RHTs and high risk imports were significantly more likely to be identified as positive reactors on RHT. Cattle were significantly more likely to be identified as inconclusive reactors when they were purchased from Scottish herds with unconfirmed inconclusive RHTs and high risk imports (OR: 1.92, 95% CI: 1.34 – 2.76, p < 0.001), Scottish herds with unconfirmed inconclusive RHTs and no high risk imports (OR: 2.25, 95% CI: 1.24 – 4.06, p = 0.007), and Scottish herds with no VETNET database entries (OR: 1.39, 95% CI: 109 – 1.78, p = 0.008).

**Table 5:** Univariate analysis of animal-level risk of being identified as a positive or inconclusive tuberculin reactor on RHT associated with previous movements

	** Positive reactor**			** Inconclusive reactor**		
**Previously located in:**	**OR**	**95% CI **	**p-value**	**OR**	**95% CI **	**p-value**
Univariate analysis						
Overseas herd	0.75	0.36 – 1.58	0.456	0.76	0.54 – 1.08	0.119
High incidence parish of England and Wales	1.31	0.63 – 2.76	0.471	0.92	0.62 – 1.37	0.692
Low incidence parish of England and Wales	-	-	-	-	-	-
Scottish herd classified as:						
confirmed positive RHT with high risk imports	1.27	0.18 – 9.21	0.813	1.52	0.67 – 3.45	0.316
confirmed positive RHT with no high risk imports	4.31	0.59 – 31.4	0.149	-	-	-
unconfirmed positive RHT with high risk imports	6.08	2.19 – 16.8	<0.001	1.60	0.65 – 3.90	0.305
unconfirmed positive RHT with no high risk imports	-	-	-	1.44	0.35 – 5.88	0.609
unconfirmed inconclusive RHT with high risk imports	0.93	0.34 – 2.56	0.884	1.84	1.28 – 2.65	<0.001
unconfirmed inconclusive RHT with no high risk imports	2.15	0.67 – 6.87	0.198	1.96	1.09 – 3.52	0.024
negative RHT and with high risk imports	0.91	0.55 – 1.49	0.704	0.78	0.61 – 0.98	0.036
negative RHT with no high risk imports	1.31	0.74 – 2.31	0.353	0.94	0.70 – 1.25	0.656
no VETNET database entries	1.15	0.69 – 1.91	0.585	1.32	1.03 – 1.68	0.025
Multivariate analysis						
Scottish herd classified as:						
unconfirmed positive RHT with high risk imports	6.28	2.26 – 17.4	<0.001			
unconfirmed inconclusive RHT with high risk imports				1.92	1.34 – 2.76	<0.001
unconfirmed inconclusive RHT with no high risk imports				2.25	1.24 – 4.06	0.007
no VETNET database entries				1.39	1.09 – 1.78	0.008

The sample was restricted to data from 2006 to 2009 and animals that were located on at least one other farm prior to testing. Both the univariate and multivariate analyses were performed using multinomial logistic regression models with age included as a confounding variable.

### Herd-level risk associated with past movement history

Results from the univariate and multivariate analyses of herd-level risks associated with past movement history are presented in Table 6. In the multivariate analysis, purchasing cattle from overseas was associated with an increased risk of confirmed positive RHT herds disclosing reactors (OR: 4.13, 95% CI: 1.47 – 11.6, p = 0.002), while purchasing cattle from unconfirmed inconclusive RHT herds with recent import movements in Scotland was a risk factor for unconfirmed inconclusive RHT herds (OR: 1.35, 95% CI: 1.03 – 1.78, p = 0.037). None of the remaining associations were significant.

**Table 6:** Univariate and multivariate analyses of the herd-level risk of having a non-negative RHT associated with cattle movements

	** Confirmed positive RHT**			** Unconfirmed positive RHT**			** Unconfirmed inconclusive RHT**		
**Purchased cattle from:**	**OR**	**95% CI **	**p-value**	**OR**	**95% CI **	**p-value**	**OR**	**95% CI **	**p-value**
Univariate analysis									
Overseas herd	4.32	1.54 – 12.2	0.005	1.01	0.47 – 2.12	0.989	1.23	0.92 – 1.64	0.167
High incidence parish of England and Wales	1.69	0.57 – 5.03	0.343	1.39	0.71 – 2.71	0.340	1.11	0.84 – 1.46	0.438
Low incidence parish of England and Wales	1.38	0.35 – 5.45	0.643	0.21	0.03 – 1.57	0.129	0.62	0.37 – 1.06	0.082
Scottish herd classified as:									
confirmed positive RHT with high risk imports	0.54	0.11 – 2.74	0.460	0.99	0.36 – 2.71	0.983	0.72	0.45 – 1.12	0.147
confirmed positive RHT with no high risk imports	1.15	0.13 – 9.85	0.901	0.78	0.10 – 6.00	0.814	0.50	0.18 – 1.38	0.180
unconfirmed positive RHT with high risk imports	0.78	0.22 – 2.76	0.700	1.28	0.59 – 2.80	0.531	1.17	0.85 – 1.62	0.331
unconfirmed positive RHT with no high risk imports	0.85	0.21 – 3.45	0.820	0.29	0.66 – 1.24	0.094	0.92	0.62 – 1.37	0.683
unconfirmed inconclusive RHT with high risk imports	1.92	0.53 – 6.95	0.323	1.79	0.89 – 3.62	0.103	1.39	1.03 – 1.80	0.028
unconfirmed inconclusive RHT with no high risk imports	0.86	0.29 – 2.56	0.792	0.90	0.46 – 1.74	0.745	1.21	0.93 – 1.57	0.158
negative RHT and with high risk imports	1.70	0.42 – 6.78	0.456	1.61	0.60 – 4.38	0.347	0.83	0.50 – 1.38	0.482
negative RHT with no high risk imports	-	-	-	0.88	0.40 – 1.93	0.751	0.85	0.62 – 1.16	0.323
no VETNET database entries	-	-	-	-	-	-	1.12	0.64 – 1.98	0.702
Multivariate analysis									
Overseas herd	4.13	1.47 – 11.6	0.002						
Scottish herd classified as:									
unconfirmed inconclusive RHT with high risk imports							1.35	1.03 – 1.78	0.037

The sample was restricted to data from 2006 to 2009. Both the univariate and multivariate analyses were performed using multinomial logistic regression models with the total number of cattle movements included as a confounding variable.

### Comparison of risk factors between confirmed positive, unconfirmed positive, unconfirmed inconclusive, and negative RHT herds

The univariate case-case comparisons revealed only a few significant differences in the demographic profiles of confirmed positive RHT herds, unconfirmed positive RHT herds, and unconfirmed inconclusive RHT herds (Table 7). In the multivariate models (Table 8), for every 1% increase in the percentage of imported cattle in the herd, the odds of being a confirmed positive RHT herd compared to an unconfirmed positive RHT herd increased by a factor of 1.12 (95% CI: 1.04 – 1.24, p = 0.013) for every 100 km increase in the northing coordinate, the odds of being a confirmed positive RHT herd decreased by a factor of 0.65 (95% CI: 0.46 – 0.90, p = 0.006). Increases in the number of farms within a 5 km radius was also associated with an increased risk (OR: 0.19, 95% CI: 0.05 – 0.61, p = 0.001). In the comparison of confirmed positive RHT herds to unconfirmed inconclusive RHT herds, the log transformed number of cattle present (OR: 2.97, 95% CI: 1.56 – 5.78, p = 0.001) and the easting coordinate (OR: 0.60, 95% CI: 0.41 – 0.89, p = 0.011) were the remaining predictor variables. For the comparison of unconfirmed positive RHT herds to unconfirmed inconclusive RHT herds, the only variable to remain significant in the multivariate analysis was the log transformed number of herds within a 5 km radius (OR: 2.61, 95% CI: 1.39 – 5.24, p < 0.001). The area under the ROC curve (AUC) was less than 0.70 for all three models indicating poor discriminatory power.

**Table 7:** Univariate case-case comparisons of demographic risk factors between confirmed positive RHT herds, unconfirmed positive RHT herds, and unconfirmed inconclusive RHT herds

	**Confirmed positive RHT to Unconfirmed positive RHT**			**Confirmed positive RHT to Unconfirmed inconclusive RHT**			**Unconfirmed positive RHT to Unconfirmed inconclusive RHT**		
**Variable**	**OR**	**95% CI **	**p-value**	**OR**	**95% CI **	**p-value**	**OR**	**95% CI **	**p-value**
log_10_ (Number of cattle tested)	1.52	0.76 – 3.13	0.247	2.24	1.19 – 4.31	0.014	1.36	0.88 – 2.12	0.170
log_10_ (Number of cattle present)	1.70	0.82 – 3.73	0.170	2.48	1.34 – 4.76	0.005	1.46	0.96 – 2.27	0.082
% of herd imported from high incidence regions	1.09	1.01 – 1.19	0.040	1.04	0.99 – 1.06	0.067	0.98	0.93 – 1.02	0.482
log_10_ (Number of cattle moved onto farm)	1.04	0.66 – 1.63	0.874	1.26	0.86 – 1.83	0.226	1.22	0.93 – 1.61	0.154
log_10_ (Number of farms within 5 km radius)	0.29	0.09 – 0.87	0.029	1.01	0.51 – 2.19	0.987	2.61	1.39 – 5.24	0.005
Neighbouring herd with:									
confirmed positive RHT	0.70	0.33 – 1.40	0.323	0.98	0.51 – 1.80	0.970	1.42	0.92 – 2.14	0.105
unconfirmed positive RHT	1.11	0.56 – 2.16	0.764	1.34	0.75 – 2.34	0.309	1.21	0.79 – 1.83	0.371
unconfirmed inconclusive RHT	0.98	0.25 – 0.96	0.955	1.15	0.63 – 2.27	0.660	1.18	0.75 – 1.91	0.488
Northing coordinate (100 km units)	0.70	0.51 – 0.95	0.025	0.73	0.57 – 0.93	0.014	0.99	0.84 – 1.17	0.924
Easting coordinate (100 km units)	0.61	0.37 – 0.97	0.041	0.71	0.49 – 1.02	0.064	1.08	0.82 – 1.43	0.579
Herd production type:									
Beef	Ref	-	-	Ref	-	-	Ref	-	-
Beef fattening	-	-	-	-	-	-	-	-	-
Beef suckler	1.15	0.56 – 2.37	0.703	1.36	0.73 – 2.53	0.331	1.18	0.76 – 1.82	0.457
Dairy	1.32	0.57 – 3.03	0.501	1.96	0.96 – 3.93	0.059	1.48	0.86 – 2.47	0.142
Mixed	-	-	-	-	-	-	-	-	-
log_10_ (Average number of sheep per year)	0.93	0.74 – 1.16	0.498	0.96	0.79 – 1.16	0.628	1.03	0.90 – 1.19	0.650
log_10_ (Average number of poultry per year)	0.90	0.32 – 2.24	0.827	0.72	0.31 – 1.34	0.369	0.78	0.45 – 1.21	0.316

The analysis included all Scottish cattle herds that were subject to RHT from 2002 to 2009.

**Table 8:** Multivariate case-case comparisons of demographic risk factors between confirmed positive RHT herds, unconfirmed positive RHT herds, and unconfirmed inconclusive RHT herds

**Variable**	**OR**	**95% CI**	**p-value**	**AUC**
Confirmed positive RHT to Unconfirmed positive RHT				
% of herd imported from high incidence regions	1.12	1.04 – 1.24	0.013	0.69
log_10_ (Number of farms within 5 km radius)	0.19	0.05 – 0.61	0.006	
Northing coordinate (100 km units)	0.65	0.46 – 0.90	0.012	
Confirmed positive RHT to Unconfirmed inconclusive RHT				
log_10_ (Number of cattle present)	2.97	1.56 – 5.78	0.001	0.64
Easting coordinate (100 km units)	0.60	0.41 – 0.89	0.011	
Unconfirmed positive RHT to Unconfirmed inconclusive RHT				
log_10_ (Number of farms within 5 km radius)	2.61	1.39 – 5.24	<0.001	0.57

The analysis included all Scottish cattle herds that were subject to RHT from 2002 to 2009.

In contrast, all three RHT case groups were significantly different from the negative RHT herds in the multinomial case–control comparison of demographic risk factors. The results from the univariate and multivariate analyses are presented in Tables 9 and 10, respectively. In general, the odds of disclosing reactors on RHT increased with the number of cattle tested, the percentage of cattle in the herd imported from high incidence regions, and when there was at least one neighbouring herd with an unconfirmed positive or inconclusive RHT result. For confirmed positive RHT herds and unconfirmed inconclusive RHT herds, the odds decreased as the total number of farms within a 5 km radius increased. The odds ratio for dairy herds changed sign between the univariate and multivariate analyses for unconfirmed inconclusive RHT herds, which suggests confounding with other model variables.

**Table 9:** Univariate case–control comparisons of demographic risk factors between confirmed positive RHT herds, unconfirmed positive RHT herds, unconfirmed inconclusive RHT herds, and negative RHT herds

	**Confirmed positive RHT**			**Unconfirmed positive RHT**			**Unconfirmed inconclusive RHT**		
**Variable**	**OR**	**95% CI **	**p-value**	**OR**	**95% CI **	**p-value**	**OR**	**95% CI **	**p-value**
log_10_ (Number of cattle tested)	9.03	4.93 – 16.5	<0.001	5.82	3.89 – 8.69	<0.001	4.38	3.75 – 5.13	<0.001
log_10_ (Number of cattle present)	7.05	3.98 – 12.5	<0.001	4.49	3.07 – 6.56	<0.001	3.22	2.78 – 3.78	<0.001
% of herd imported from high incidence regions	1.05	1.02 – 1.07	<0.001	1.02	0.99 – 1.06	0.220	1.03	1.02 – 1.04	<0.001
log_10_ (Number of cattle moved onto farm)	2.05	1.48 – 2.85	<0.001	1.99	1.58 – 2.51	<0.001	1.70	1.55 – 1.86	<0.001
log_10_ (Number of farms within 5 km radius)	0.75	0.39 – 1.45	0.401	1.70	0.96 – 3.02	0.068	0.75	0.62 – 0.91	<0.001
Neighbouring herd with:									
confirmed positive RHT	1.40	0.77 – 2.55	0.274	2.00	1.36 – 2.96	<0.001	1.42	1.19 – 1.68	<0.001
unconfirmed positive RHT	1.97	1.14 – 3.41	0.015	1.78	1.20 – 2.64	0.004	1.47	1.24 – 1.73	<0.001
unconfirmed inconclusive RHT	2.15	1.16 – 3.98	0.015	2.19	1.42 – 3.40	<0.001	1.86	1.58 – 2.20	<0.001
Northing coordinate (100 km units)	0.58	0.46 – 0.73	<0.001	0.76	0.66 – 0.88	<0.001	0.77	0.73 – 0.81	<0.001
Easting coordinate (100 km units)	0.80	0.58 – 1.09	0.165	1.13	0.89 – 1.43	0.299	1.06	0.97 – 1.17	0.205
Herd production type:									
Beef	Ref	-	-	Ref	-	-	Ref	-	-
Beef fattening	-	-	-	-	-	-	0.51	0.24 – 1.09	0.083
Beef suckler	1.30	0.72 – 2.35	0.386	1.13	0.75 – 1.70	0.553	0.96	0.82 – 1.13	0.614
Dairy	2.60	1.33 – 5.09	0.005	1.96	1.21 – 3.18	0.007	1.33	1.08 – 1.64	0.009
Mixed	-	-	-	-	-	-	5.58	1.43 – 21.7	0.013
log_10_ (Average number of sheep per year)	1.14	0.94 – 1.39	0.190	1.25	1.08 – 1.44	0.002	1.21	1.14 – 1.28	<0.001
log_10_ (Average number of poultry per year)	0.48	0.23 – 1.02	0.058	0.53	0.32 – 0.88	0.013	0.71	0.60 – 0.84	<0.001

The analysis included all Scottish cattle herds that were subject to RHT from 2002 to 2009 and used multinomial logistic regression models with herd bTB risk classification as the outcome variable.

**Table 10:** Multivariate case–control comparison of demographic risk factors between confirmed positive RHT herds, unconfirmed positive RHT herds, unconfirmed inconclusive RHT herds, and negative RHT herds

	**Confirmed positive RHT**			**Unconfirmed positive RHT**			**Unconfirmed inconclusive RHT**		
**Variable**	**OR**	**95% CI **	**p-value**	**OR**	**95% CI **	**p-value**	**OR**	**95% CI **	**p-value**
log_10_ (Number of cattle tested)	8.56	4.59 – 16.0	<0.001	5.78	3.77 – 8.87	<0.001	4.72	3.98 – 5.58	<0.001
% of herd imported from high incidence regions	1.05	1.02 – 1.08	<0.001				1.03	1.01 – 1.04	<0.001
log_10_ (Number of farms within 5 km radius)	0.24	0.11 – 0.56	<0.001				0.38	0.30 – 0.48	<0.001
Neighbouring herd with:									
unconfirmed positive RHT	1.79	0.99 – 3.20	0.050				1.37	1.15 – 1.64	<0.001
unconfirmed inconclusive RHT	2.12	1.03 – 4.31	0.040	1.65	1.03 – 2.63	0.037	1.90	1.57 – 2.31	<0.001
Herd production type:									
Beef							Ref	-	-
Dairy							0.70	0.55 – 0.88	0.003

The analysis included all Scottish cattle herds that were subject to RHT from 2002 to 2009 and used a multinomial logistic regression model with herd bTB risk classification as the dependent variable.

Overall, there was a strong positive correlation between the total number of cattle present on the farm and the number of individual RHTs performed between 2002 and 2009 (the Pearson correlation coefficient r = 0.88, p < 0.001). There was a moderate positive correlation between the total number of cattle present on the test date and the total number of cattle moved onto the farm in the 4 years prior (r = 0.72, p < 0.001).

### Change in herd-level risk of disclosing at least one tuberculin reactor over time

**Figure 3:**
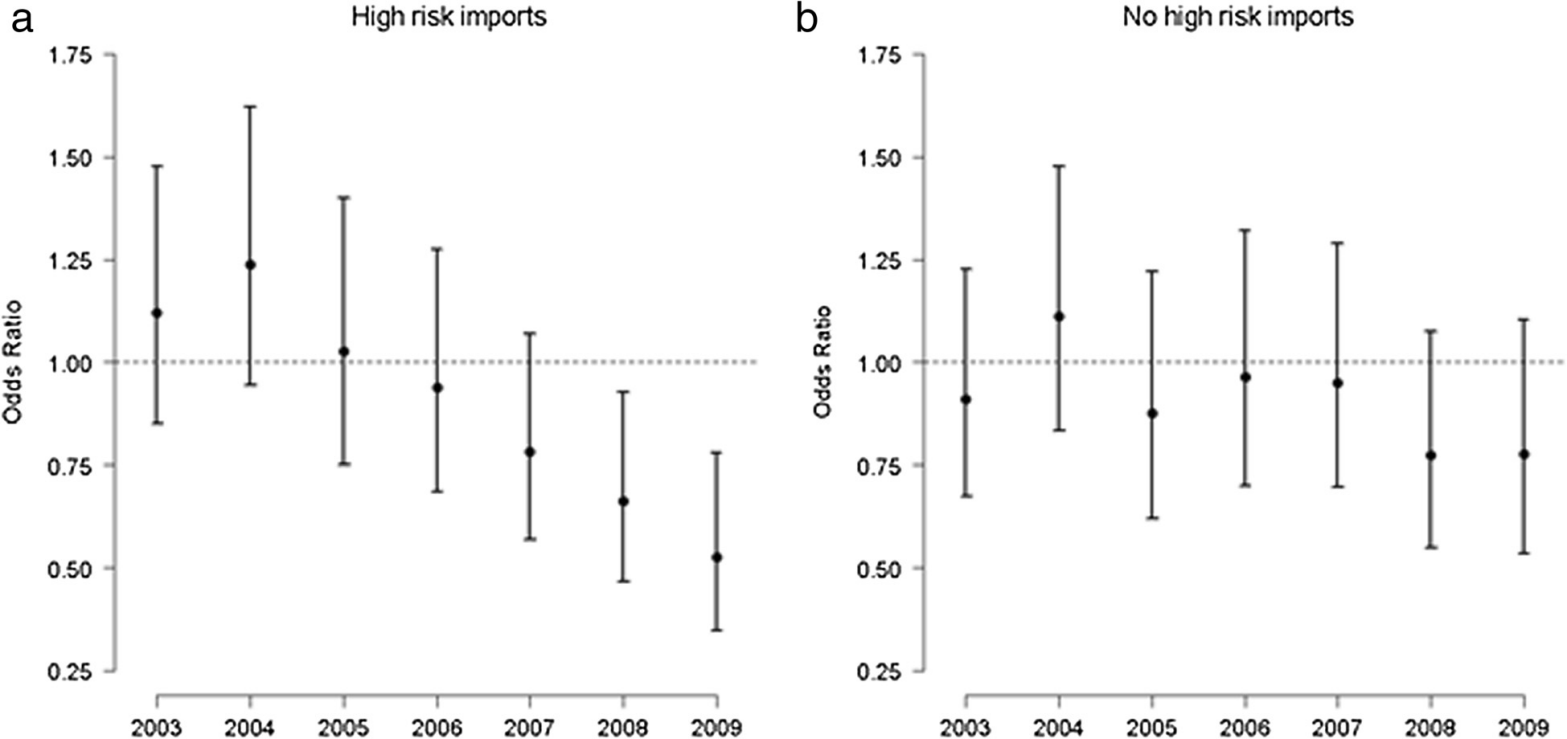
**Change in the odds of Scottish farms (a) with and (b) without recent high risk imports disclosing at least one tuberculin reactor through RHT from 2002 to 2009.** The point estimates and vertical bars correspond to the predicted odds ratios (OR) and 95% confidence intervals (CIs) from a mixed-effects logistic regression model with the year of testing as the fixed effect and farm CPH as the random effect. The reference year is 2002.

As shown in Figure 3a, there was a significant downward trend over time in the odds of farms with recent high risk imports having at least one animal identified as a positive or inconclusive reactor through routine surveillance. Using 2002 as the baseline year, farms with recent high risk imports that were tested in 2009 were 0.53 times as likely to have at least one reactor compared to herds that were tested in 2002 (95% CI: 0.34 – 0.78, p < 0.001). There was no noticeable trend in the risk for herds without recent high risk imports from 2002 to 2009 (Figure 3b).

## Discussion

The two main objectives of this study were to investigate the risks of bTB associated with cattle movements and to identify risk factors that may aid in the interpretation of unconfirmed positive or inconclusive RHT results. Overall, one of the most important findings was that the majority of confirmed positive RHT and slaughter cases in Scotland were triggered by cattle that were located exclusively on Scottish cattle farms prior to the detection date. This provides indirect evidence that bTB transmission occurred within Scotland during the study time period. In most cases, these farms had a history of importing cattle from high risk regions, which may have originally been responsible for introducing bTB. Other cases of on-farm transmission have been documented in the literature [27]. Fischer and colleagues, for example, describe an outbreak in the Netherlands where the import of an infected animal generated a single additional reactor detected 392 days later [21]. In a New Zealand dairy herd, the purchase of a single infected animal resulted in eight further confirmed reactors identified over a 2-year period [20]. With the high frequency of movements between Scottish cattle farms [28], there is a danger that the infected animals will be sold to other herds leading to secondary bTB outbreaks prior to detection through routine surveillance. Based on findings from the animal- and herd-level risk factor analyses as well as the case-case and case–control comparisons, we also cannot rule out the possibility that some of the herds with unconfirmed positive or inconclusive RHT results were truly infected with bTB and at risk of spreading disease to other cattle farms.

### Animal and herd-level risks associated with past movement history

In the animal-level risk factor analysis, cattle that were purchased from Scottish herds with unconfirmed positive RHTs and high risk imports were at significantly increased risk of being identified as positive reactors on RHT, while cattle that were purchased from unconfirmed inconclusive RHT herds with or without high risk imports were at significantly increased risk of being identified as inconclusive reactors. Other case–control studies from bTB endemic regions have similarly shown that cattle purchased from herds with positive reactors were significantly more likely to react on subsequent herd tests in the receiving herds [29,30]. It is therefore possible that these Scottish source herds were serving as a reservoir of bTB or other potentially cross-reacting mycobacterial antigens. Based on results from the herd-level analysis showing that purchasing imported cattle was a significant risk factor for confirmed positive RHT herds, we would also expect that unconfirmed positive herds with a history of importing high risk cattle are less likely to be false positives than those without high risk imports. One possible explanation for why unconfirmed inconclusive herds without high risk import movements was a risk factor is that these were generally not placed under movement restrictions or subject to follow-up testing, which increases the chance that any truly positive herds will have the opportunity to spread bTB through cattle movements. Additional considerations include the possibility that our movement history variables are proxies for other herd management practices that increase the risk of bTB and the possibility that regulatory officials are more likely to classify an animal as a positive or inconclusive reactor if there is reason to suspect potential exposure to bTB infected cattle. However, testing these hypotheses was beyond the scope of the current study.

### Comparison of risk factors between confirmed positive, unconfirmed positive, unconfirmed inconclusive, and negative RHT herds

Herds with unconfirmed positive and inconclusive reactors represent a particular regulatory challenge since the costs associated with cattle movement restrictions, contact tracing, and follow-up short interval tests can be significant for both the farmer and the taxpayer [31] and these herds outnumber confirmed positive RHT cases by a factor of almost 20. The case-case comparisons revealed that the confirmed positive RHT herds were virtually indistinguishable from unconfirmed positive and unconfirmed inconclusive RHT herds with a few notable exceptions. The confirmed positive RHT herds had a greater percentage of imported cattle, fewer farms within a 5 km radius, and were more likely to be located in the south of Scotland compared to unconfirmed positive RHT herds. The confirmed positive RHT herds were significantly larger and more likely to be located in the west than unconfirmed inconclusive RHT herds. These variables may be proxies for the intensity of exposure to imported cattle.

As expected in the case–control comparisons, the total number of cattle tested during RHT was one of the strongest risk factors for having non-negative RHT results, but this was also highly correlated with the total number of cattle present in the herd from 2002 to 2009 and moderately correlated with the total number of cattle moved onto the farm in the 4 years prior to RHT. It is therefore difficult to determine whether large herds are at risk simply because of surveillance design [8,18] or whether there are other management factors, such as higher stocking densities, greater volumes of cattle movements, and heavier environmental contamination, that predispose large herds to having more frequent or more severe bTB outbreaks [32-34]. The most likely explanation for the protective effects of having a larger number of farms within a 5 km radius is that larger herds are more likely to have fewer neighbours since the farm occupies a greater area of land surrounding the main farm point location.

Herds with at least one neighbouring farm that disclosed unconfirmed positive or inconclusive reactors through RHT during the study period were generally more likely to have non-negative RHT results themselves. Evidence of spatial association in confirmed tests results have been reported in other studies [9,35,36]. There are several possible explanations for these findings. First, neighbouring herds may be more likely to purchase cattle from similar sources or trade directly with each other. Second, there may be other cross-reacting mycobacterial antigens in the production environment, such as *M. avium* subsp. *paratuberculosis*[37]*or M. caprae*[25], that can lead to false positive reactions to SICCT in exposed cattle. Third, SICCT interpretation is relatively subjective and veterinarians may be more likely to classify an animal as a positive or inconclusive reactor if there is a history of bTB in neighbouring herds [38]. Finally, the presence of wildlife reservoirs for bTB within the local farm environment cannot be ruled out although no bTB infections have been reported in Scottish wildlife.

The significance of herd production type was more difficult to interpret given the evidence of confounding in the multivariate models. In the comparison between unconfirmed inconclusive RHT herds and negative herds, dairy herds were at increased risk in the univariate analysis, but at decreased risk in the multivariate analysis. Herd type was most likely confounded with the easting and northing coordinates as most large dairy farms in Scotland are located in the south-west. Amongst other published studies, there is considerable disagreement in the role of herd production type in the epidemiology of bTB [36,39-41]. Altogether, the case-case and case–control comparisons highlight the difficulties in using demographic risk factors to determine whether unconfirmed herds are truly infected with bTB.

### Change in herd-level risk of disclosing at least one tuberculin reactor or bTB lesioned animal over time

The observed decrease in the risk of identifying reactors or lesioned cattle over time provides additional support for the hypothesis that many confirmed bTB cases in Scotland were attributable to cattle imported from high risk regions. Following the introduction of post-movement testing in 2005, Gates and colleagues showed that the volume of cattle imported from high incidence parishes decreased substantially as the result of changes in farmer trade behaviour, which may have reduced the risk of disease introduction as much, if not more, than the testing itself [19]. After the movement restrictions triggered by the foot-and-mouth disease outbreak were lifted in November 2001, there was a transient surge in cattle movements as farmers replaced animals that were culled as part of disease eradication measures. This re-stocking has previously been linked to an increased incidence of confirmed bTB cases in other regions of Great Britain [42] and it is likely that many of these imported cattle were responsible for seeding bTB in Scottish cattle herds early in the study time period. We would expect the decline in risk to continue over time as more subclinically infected cattle are removed from the Scottish population through routine culling and surveillance. Overall, this trend is encouraging from a disease control perspective.

### Study limitations

Our method of classifying Scottish herds into risk categories was simply based on the presence or absence of imported cattle and the aggregate routine surveillance results over the study time period. We recognize that other factors such as the number of cattle imported by the farm, the length of time imported animals previously spent in high-risk regions, and the length of time imported animals subsequently spent on the receiving farm were also likely important in determining the risk of bTB being present in the herd. Further investigation into these risk factors may provide additional insight into the types of herds that are worth targeting with increased surveillance, and the management factors that increase the possibility for bTB transmission within Scotland. For simplicity, we also used the PTIs from 2007 to categorize import movements as high risk, though there were some changes in PTI designation, and in the underlying incidence of bTB, during the period of study.

There were additional challenges in using the CTS database to trace the movement history of individual cattle and herds. First, several of the study farms were excluded from the analysis because the CPH code could not be linked to a valid CTS location identifier or no cattle were present on the location at the time of testing (Table 1). Second, surveillance results in the VETNET database are stored under the main farm CPH number regardless of whether cattle are housed on that location or on other uniquely identified land parcels operated by the same cattle business [43]. However, farmers that have registered for ‘linked holding’ status are not required to report the movements of cattle between land parcels belonging to their business. This may have resulted in underestimation of the exposure to imported cattle or cattle moved from non-negative Scottish herds. Given that being previously located in a herd with no recorded surveillance results was a significant risk factor in the animal-level analysis, these locations warrant further investigation. Finally, cattle that were born prior to January 2001 when farmers were first required to report movements centrally may have incomplete movement history records. It is therefore possible that a small number of older animals classified as Scottish cattle in our analysis were previously located in endemic regions of England and Wales.

## Conclusions

The eradication of bTB in Scotland remains an ongoing challenge due to limitations in the diagnostic tests used to identify and confirm bTB infections in cattle herds. Based on our study findings, we cannot rule out the possibility that bTB infected cattle go undetected and may be at risk of spreading disease within and between Scottish cattle herds. However, it is unclear whether increasing surveillance intensity would benefit disease control efforts given that the risk of disclosing reactors appears to be decreasing over time, most likely in response to the decline in high risk cattle import movements. Further investigation is warranted to determine whether targeting herds with unconfirmed reactors and a history of importing cattle from high risk regions is a cost-effective use of limited control resources.

## Competing interests

The authors declare that they have no competing interests.

## Authors’ contributions

MCG designed the study, processed the data, performed the statistical analyses, and drafted the manuscript. VVV collated the data and helped writing the manuscript. VVV and MEJW participated in the study design and interpretation. All authors have read and approved the final manuscript.
